# POLE2 silencing inhibits the progression of colorectal carcinoma cells via wnt signaling axis

**DOI:** 10.1080/15384047.2024.2392339

**Published:** 2024-08-18

**Authors:** Weihua Jian, Lei Zhang

**Affiliations:** a Department of General Surgery, The First Affiliated Hospital, Jinan University, Guangzhou, Guangdong, China; b Department of General Surgery, Guangzhou First People’s Hospital, School of Medicine, South China University of Technology, Guangzhou, China; c Department of Second General Surgery, Guangdong Second Provincial General Hospital, Guangzhou, China

**Keywords:** Colorectal cancer, POLE2, Wnt/β-catenin, proliferation, invasion

## Abstract

Colorectal cancer (CRC) is one of the most common malignant carcinoma worldwide. DNA polymerase epsilon 2, accessory subunit (POLE2) participates in DNA replication, repair, and cell cycle control, but its association with CRC development remains unclear. In the present study, the differentially expressed genes (DEGs) in CRC were screened from bioinformatics analysis based on GEO database. RT-qPCR was used to assess mRNA expression. CCK-8 and colony formation assays were applied for the evaluation of cell proliferation. Wound healing and transwell assays were used to detect cell migration and invasion. Protein levels were determined by Western blotting assay. We found that POLE2 was highly expressed in CRC tissues and cell lines. Inhibition of POLE2 suppressed the proliferation, migration and invasion of CRC cells. Mechanistically, Wnt/β-catenin signaling pathway was inactivated by inhibition of POLE2. Activation of Wnt/β-catenin pathway can reverse the function of POLE2 knockdown on CRC cells. *In vivo* studies demonstrated that POLE2 silencing could notably inhibit the growth of tumors, which was consistent with the results *in vitro*. In conclusion, we found POLE2 as a novel oncogene in CRC, providing a potential therapeutic or diagnostic target in CRC.

## Introduction

Colorectal cancer (CRC) has become the most common gastrointestinal malignant tumor in the world, with increasing incidence and mortality.^1^ The main causes of poor prognosis of CRC are postoperative recurrence and metastasis.^2^ However, most patients with CRC are diagnosed at an advanced stage, missing the optimal time for treatment. Hence, it is urgent to extend our understanding of the mechanisms of CRC progression and to provide new potential therapeutic or diagnostic targets.

In recent years, abnormal gene expression in various tumors has been found and they were further proved to participate in the regulation of malignant behaviors.^3–5^ Studies of Belhadj et al.,^6^ and Terradas et al.,^7^ declared that DNA polymerase epsilon 2, accessory subunit (POLE2) is a differentially expressed gene in CRC. POLE2 is a subunit of the eukaryotic DNA polymerase epsilon.^8^ POLE2 participates in the regulation of diverse cellular functions, including modulating cell proliferation.^9^ Moreover, aberrant expression of POLE2 has been reported in breast, colorectal, and cervical cancers.^10–12^ However, the underlying mechanism of the effect of POLE2 in the development of CRC remains to be elucidated.

The purpose of this study was to investigate the role of POLE2 in the proliferation, migration and invasion of CRC cells. *In vivo* studies were carried out to further confirm the effect of POLE2. In addition, the molecular mechanism of POLE2 in CRC was also discussed. We hope these findings will provide novel targets for the treatment and diagnosis of CRC.

## Materials and methods

### Bioinformatic analysis

The expression of IL12RB1, POLE2, MRE11 and POT1 genes in CRC cancer and its adjacent tissues was analyzed in GEPIA (http://gepia.cancer-pku.cn/.),^13^ and the expression of POLE2 in CRC was predicted by Starbase (https://rnasysu.com/encori/).

### Patients

Carcinoma and adjacent tissues were gathered from CRC patients (*n* = 28) at Guangdong Second Provincial General Hospital (2021-SB-110). The study was approved by the Ethics Committee of Guangdong Second Provincial General Hospital. Every participator have signed an informed consent form. Tissues collected from all patients who had not received chemotherapy or radiotherapy before surgery were rapidly frozen in liquid nitrogen and stored in a −80°C environment.

### Cell culture

Human CRC RKO (CRL-2577), SW480 (CCL-228), HCT116 (CCL-247), SW620 (CCL-227), LoVo (CCL-229) cells and human normal colon epithelial cell FHC (CRL-1831) cells were all purchased from ATCC (Manassas, VA). FHC cells were cultured in RPMI 1640 (Thermo Fisher Scientific, USA) with 10% FBS (Gibco, Waltham) and 1% penicillin/streptomycin (Gibco, Waltham).^14^ CRC cell lines were maintained in DMEM (Thermo Fisher Scientific, USA) with 10% FBS. LiCl (20 mM 793,620, Sigma-Aldrich, USA) is a glycogen synthase kinase-3beta (GSK3β) inhibitor that activates Wnt signaling, and was used to treat cells for 48 h.^15^

### Target gene RNA interference

The short hairpin RNA of POLE2 (sh-POLE2) and its negative control (sh-control) were designed by Shanghai Gene Chem Co, Ltd. (China) to silence POLE2. These shRNAs were transfected into RKO and SW480 cells using Lipofectamine 3000 (Invitrogen, Carlsbad, CA, USA) following the protocols.

### Real-time quantitative PCR (rt-qPCR)

Total RNA was extracted with TRIzol reagent (Invitrogen). Total RNA (1 μg) was reverse transcribed into cDNAs using a Transcriptor cDNA Synth. Kit (Roche, Germany). Afterward, the PCR reaction was carried out in a FAST7500 system (ABI, USA) with a SYBR Ggreen Mix I kit (Jiancheng, China). The relative expression was calculated by 2^−∆∆CT^ method. The primer sequences used were as follows:

POLE2: F: 5′-TGAGAAGCAACCCTTGTCATC-3′, R: 5′-TCATCAACAGACTGACTGCATTC-3′

IL12RB1: F: 5′-TAGGGACCTGAGATGCTATCG-3′, R: 5′-CCCGGAGCTAAGGCAACAC-3′

MRE11: F: 5′-ATGCAGTCAGAGGAAATGATACG-3′, R: 5′-CAGGCCGATCACCCATACAAT-3′

POT1: F: 5′-CAGCCAATGCAGTATTTTGACC-3′, R: 5′-GGTGCCATCCCATACCTTTAGAA-3′

GAPDH: F: 5’-GAGTCCACTGGCGTCTTCAC-3’, R: 5’-ATCTT GAGGCTGTTGTCATACTTCT-3’.

### Western blot

The RKO and SW480 cells or tumor tissues were collected and lysed with RIPA lysis buffer (Cell Signal Technology, Danvers, MA, USA). Protein concentrations were detected with a BCA protein assay Kit (23225, HyClone-Pierce, Waltham, MA, USA). Each protein (20 µg) was transferred to PVDF membrane, and then incubated overnight at 4°C with the following primary antibodies: anti-POLE2 (PA5–36952, 1:500; Invitrogen), anti-E-cadherin (PA5–32178, 1:500; Invitrogen), anti-vimentin (PA5–27231, 1:800; Invitrogen), anti-N-cadherin (PA5–17526, 1:800; Invitrogen), anti-PI3K (ab191606, 1:1000; Abcam), anti-p-PI3K (ab182651, 1:500; Abcam), anti-AKT (ab233755, 1:1000; Abcam), anti-p-AKT (ab38449, 1:1000; Abcam), anti-p38 (GTX110720, 1:500; GeneTex), anti-p-p38 (ab195049, 1:1000; Abcam), anti-GSK3β (ab32391, 1:5000; Abcam), anti-p-GSK3β (ab75814, 1:10000; Abcam), anti-β-catenin (ab32572, 1:5000; Abcam) and GAPDH (MA5–15738-D680, 1:3000; Invitrogen) antibody. Afterward, blots were then incubated with a horseradish peroxidase (HRP) conjugated goat anti-rabbit IgG polyclonal secondary antibody (A27036, 1:4000; Invitrogen) for 1 h. Immunoreactive blots were viewed by ECL and plus TM western blotting system kit (RPN2232, Amersham, Chalfont, UK) and gray value of the bands was quantified using Image J software.

### Phospho-kinase array

Human Phospho-Kinase Array kit purchased from R&D were used to analyze the phosphorylation profiles of kinases and their protein substrates. Briefly, protein concentration in the supernatants of RKO cells was quantified, and the cell lysates were incubated with the microarrays at 4°C overnight. After mixture with biotin-labeled antibodies for 2 h, microarrays were incubated with HRP-streptavidin for 30 min at room temperature, and then detected using a western blot assay. A signal was produced at each capture spot corresponding to the amount of a phosphorylated protein bound.

### CCK-8 method

100 μL cell suspension (2 × 10^4^ cells/mL) were seeded into 96-well plates. The plates were pre-cultured in an incubator for 24 h (37°C, 5% CO_2_). CCK-8 solution (10 μL, AmyJet Technology, China) was added to each well and incubated with cells for 1 h. The absorbance at 450 nm was measured with a microplate reader (Nanjing DeTie Experimental Equipment Co., Ltd., China).

### Colony formation assay

The RKO and SW480 cells were inoculated into 6-well plates (1000/well) and cultured for 14 days. Thereafter, the cells were fixed in 4% paraformaldehyde for 15 min and then stained with crystal violet (0.1%) for 10 min. Colonies were photographed and the numbers were counted.

### Transwell assay

Transwell chambers (24-well, 8-mm pore) (Corning, USA) were seeded onto an empty 24-well plate and cultured at 37°C for 2 h. The RKO and SW480 cells were digested, and then suspended in a serum-free medium. The 100 μL cell suspension (1 × 10^6^ cells/mL) was loaded into the upper chamber of the transwell and the lower chamber of the transwell was filled with 500 μL culture mediums containing 20% FBS for incubation 48 h at 37°C. As for invading cell count, matrigel (Corning Inc., USA) was precoated in the upper chamber. The cells adhering to the membrane were fixed in 4% paraformaldehyde for 30 min and stained with 0.1% crystal violet for 20 min. Finally, five fields of each well were randomly selected under the microscope (Nikon, Tokyo, Japan).

### CRC transplanted tumor model

SPF male BALB/C nude mice (*n* = 6) were purchased from Huaxing Experimental Animal Farm, Huiji District, Zhengzhou City. The certificate number of experimental animal was SCXK(Yu)2019–0002. The mice were randomly divided into two groups. 0.2 mL RKO-sh-POLE2 and RKO-sh-Control cell suspension (1 × 10^7^ cells/mL) was subcutaneously injected into the dorsal part of the armpit of BALB/C nude mice. After tumor cells were inoculated, the diet, mental and activity status of nude mice were traced. During the experiment, the weight of nude mice, the long diameter (a) and short diameter (b) of the transplanted tumor were measured. The volume of the transplanted tumor was calculated according to the volume formula V = 1/2 (ab^2^),^16^ and measured every 3 days after modeling.

### Immunohistochemistry

After embedded in paraffin, tumor samples were sectioned, deparaffinized, and rehydrated. Antigens were then retrieved with sodium citrate buffer (10 mM sodium citrate, 0.05% tween 20, pH 6.0), and the sections were treated with 3% H_2_O_2_ and blocked with 5% goat serum. The sections were then incubated with anti-POLE2 (PA5–103788, 1:100; Invitrogen) or anti-Ki67 (PA1–21520, 5 µg/mL; Invitrogen) overnight at 4°C, followed by incubation with Goat Anti-Rabbit IgG-HRP Conjugate (31460, Invitrogen). The sections were further counterstained with hematoxylin. Images were acquired using a microscope.

### Statistical analysis

Data were analyzed using GraphPad 8.3 and expressed as the mean ± standard deviation. Differences were analyzed using Student’s t-test and one-way analysis of variance followed Tukey-test. Differences were deemed as statistically significant at *p* < .05.

## Results

### POLE2 is a highly expressed gene in CRC

**Figure 1.**
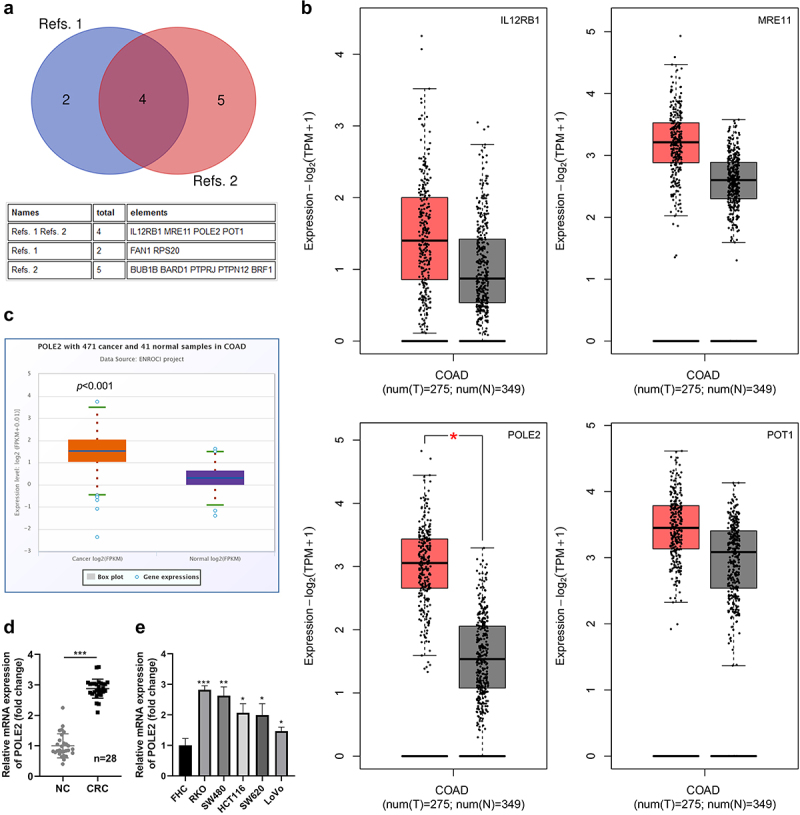
POLE2 is a highly expressed gene in colorectal cancer (CRC). (a) Venn diagram of genes identified to participate in progress of CRC. (b) IL12RB1, POLE2, MRE11 and POT1 expressions in colon adenocarcinoma (COAD) tissues from GEPIA database. (c) POT1 expressions in COAD tissues from StarBase database. The mRNA levels of POLE2 in (d) CRC tissues and (e) cell lines were evaluated by rt-qPCR. *p < .05, **p < .01, ***p < .001 vs NC or FHC group.

In the study by Belhadj et al., six genes were identified as significantly up-regulated in CRC (Refs.1),^6^ and in the study by Terradas et al., nine genes were identified as significantly up-regulated in CRC (Refs.2).^7^ As shown in the Venn diagram of Figure 1a, IL12RB1, POLE2, MRE11 and POT1 were predicted to be up-regulated in CRC in both studies. Among them, the data in GEPIA indicated that POLE2 level was more than 2.6 folds in CRC tissues as much as that in the normal tissues (Figure 1b). Moreover, POLE2 is dramatically up-regulated in CRC tumor tissues compared with normal controls according to the StarBase analysis (Figure 1c). We found that POLE2 expression in collected CRC samples was in consistent with these bioinformatic analysis (Figure 1d), and the difference in the expression of POLE2 in CRC cancer and adjacent tissues was the most significant compared with the other three genes (Figure S1A). POLE2 was also detected in normal colon epithelial cells FHC and CRC cell lines, the results demonstrated that POLE2 expression was higher in CRC cell lines than normal cells (Figure 1e, Figure S1B). RKO and SW480 cells with the highest expression level of POLE2 were selected for subsequent experiments.

### Knockdown of POLE2 inhibits cell the proliferation, migration and invasion of CRC cells

**Figure 2.**
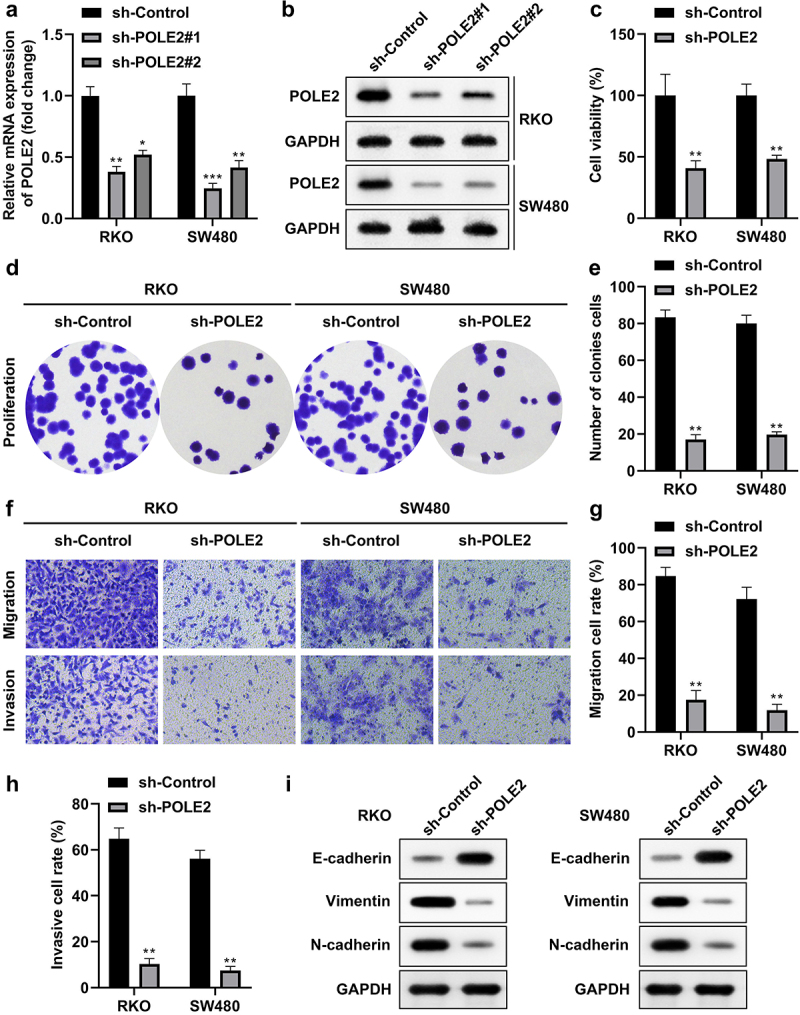
Knockdown of POLE2 inhibits cell proliferation, migration and invasion of CRC cells. (a) mRNA and (b) protein expression of POLE2 in RKO and SW480 cells after transfection were detected by qPCR and western blot, respectively. (c) CCK-8 and (d–e) colony formation assays were combined to evaluate cell proliferation. (f-h) transwell assay was performed to determine cell invasion and migration. (i) The protein expression of vimentin, N-cadherin and E-cadherin was evaluated by western blot. *p < .05, **p < .01, ***p < .001.

Then, we silenced POLE2 to check if it involves in the malignant behaviors of CRC cells. The expression level of POLE2 was prominently decreased compared with the control shRNA (sh-control), indicating the success of transfection and sh-POLE2#1 was used in the following experiments (Figure 2a,b, Figure S2A,B). Inhibition of POLE2 observably decreased the cell viability (Figure 2c, Figure S2C) and cloning formation ability of RKO and SW480 cells (Figure 2d,e, Figure S2D,E). Moreover, the migration and invasion of RKO and SW480 cells were retarded after POLE2 knockdown (Figure 2f–h, Figure S2F–H). Meanwhile, the protein expression of vimentin and N-cadherin decreased whereas E-cadherin increased (Figure 2i, Figure S2I).

### POLE2 promotes epithelial-mesenchymal transition (EMT) in CRC cells through the Wnt/β-catenin signaling pathway

**Figure 3.**
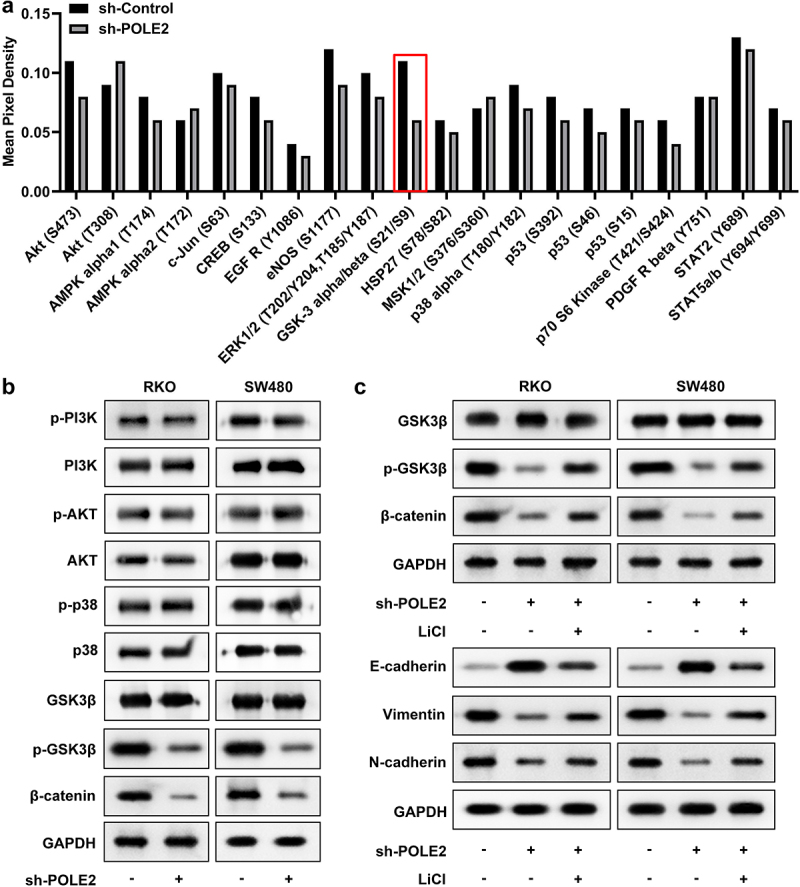
POLE2 knockdown inhibited the activation of Wnt/β-catenin pathway. (a) The cell lysates of RKO cells before and after transfection were analyzed using the human phospho-kinase array. (c, d) the activation of PI3K/AKT, p38 MAPK, and Wnt/β-catenin pathways was evaluated by western blot.

We used proteome profiler human phospho-kinase array kit to reveal possible phosphorylated kinases and signaling nodes that POLE2 affected in order to investigate the underlying mechanism. The results showed that the inhibition of POLE2 specifically down-regulated the phosphorylation of GSK3β (Figure 3a). Subsequently, to determine the mechanism underlying the oncogenic function of POLE2, the activation of several pathways were investigated by western blot assay. We can see that the phosphorylation of p-GSK3β and the level of β-catenin were significantly reduced after POLE2 knockdown compared with the sh-Control groups (Figure 3b), while the PI3K/AKT and p38 MAPK pathways were not significantly affected. These results illustrated that Wnt/β-catenin signaling pathway was inactivated by inhibition of POLE2. Meanwhile, LiCl, a GSK3β inhibitor was used to activate Wnt/β-catenin pathway. The results revealed that LiCl dramatically upregulated vimentin, N-cadherin and E-cadherin protein levels which were reduced by sh-POLE2 (Figure 3c).

### POLE2 knockdown inhibits the aggressive behaviors of CRC cells through the Wnt/β-catenin signaling pathway

**Figure 4.**
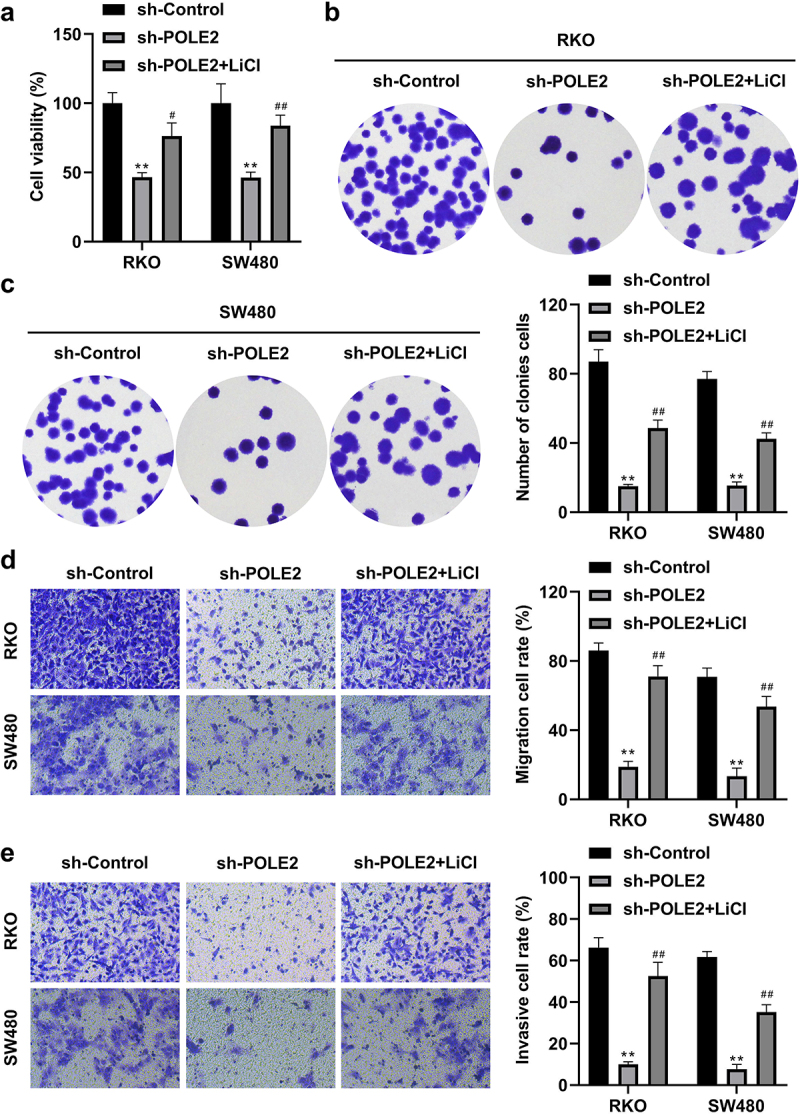
POLE2 promotes the aggressive behaviors of CRC cells through the Wnt/β-catenin signaling pathway. (a) CCK-8 and (b-c) colony formation assays were combined to evaluate cell proliferation. (d-e) transwell assay was performed to determine cell invasion and migration. **p <.01 vs sh-control group, #*p* <.05 vs sh-POLE2 group, ##*p*<.01 vs sh-POLE2 group.

Knockdown of POLE2 observably decreased the cell proliferation (Figure 4a–c), migration (Figure 4d), and invasion (Figure 4e) of RKO and SW480 cells. However, LiCl dramatically reduced the effects of POLE2 silencing on cell proliferation, invasion, and migration. These data suggested that activation of Wnt/β-catenin signaling pathway dramatically alleviated the aggressiveness of CRC cells induced by POLE2 knockdown, which means POLE2 exerts its role in CRC partially through Wnt/β-catenin pathway.

### Knockdown of POLE2 suppresses tumor growth of CRC in vivo

**Figure 5.**
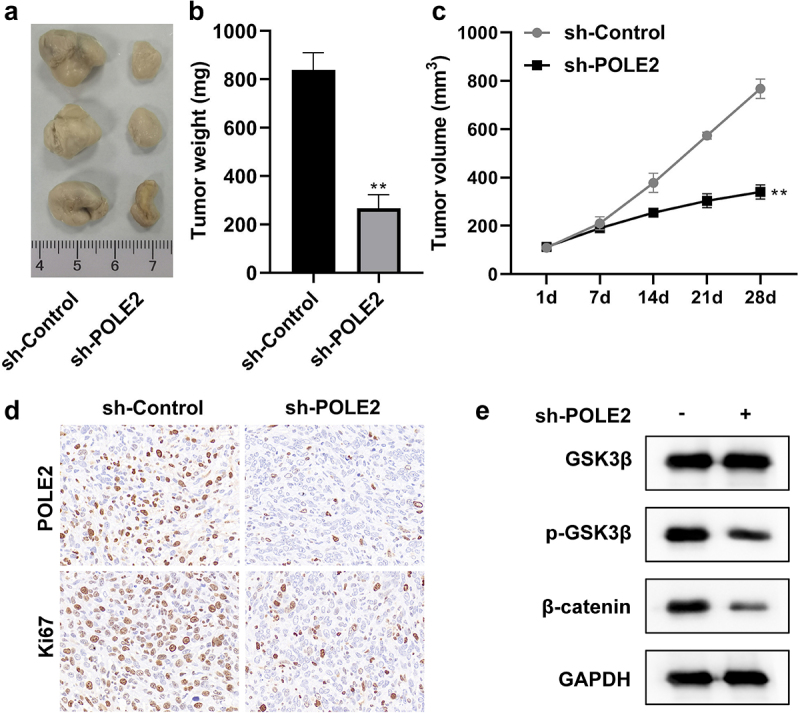
Knockdown of POLE2 suppressed tumor growth of CRC in vivo. (a) The photographs, (b) tumor weight, and (c) tumor volume of xenograft tumors from control, knockdown of POLE2 groups. (d) Representative immunohistochemistry staining images of tumors indicated the levels of POLE2 and Ki67. (e) The activation of Wnt/β-catenin pathway was evaluated by western blot. **p <.01 vs sh-control group.

Next, we performed animal experiments to clarify the functional role of POLE2 in cell growth of CRC *in vivo*. As shown in Figure 5a, tumors in the control group were larger than those in mice inoculated with POLE2-downregulated RKO cells (Figure 5a). Silencing of POLE2 leads to a remarkable diminution of tumor weight (Figure 5b). In addition, the tumor growth curves affirmed that the tumor volume of the sh-POLE2 group was significantly smaller than that of the control group (Figure 5c). Immunohistochemistry of tumor tissues showed that low protein expression of POLE2 was correlated with low protein expression of Ki67 (Figure 5d). These results of western blot also illustrated that Wnt/β-catenin signaling pathway was inactivated by inhibition of POLE2 *in vivo*.

## Discussion

At present, the molecular mechanism of POLE2 in CRC is not clear. In this study, we confirmed that POLE2 was up-regulated in CRC tissues and cell lines, indicating its carcinogenic role in CRC, which is consistent with the previous reports.^17^ Loss of function studies further demonstrated that the proliferation, migration and invasion ability of CRC cells were weakened after POLE2 deletion. These results confirmed the carcinogenic role of POLE2 in CRC. However, its mechanism needs to be further elucidated.

To figure out the signaling pathways through which POLE2 functions. Several pathways of tumor cell proliferation, migration and invasion were selected, and the expression of proteins in these pathways was detected by western blot. We found that knocking down of POLE2 inhibited the activation of Wnt/β-catenin pathway.

The Wnt/β-catenin signaling pathway is inactive in normal cells and has multiple action sites.^18^ Abnormal activation of Wnt/β-catenin signaling is an important cause of CRC.^17^ Binding of Wnt to receptor can inhibit the formation of β-catenin-degradation complex. The β-catenin degradation barrier aggregates in the cytoplasm, and translocates into the nucleus, binds to the corresponding transcription factor T-cell factor/lymphoid enhancer factor (TCF/LEF) to activate transcription of target genes and promote proto-oncogene expression.^19^ Tumor-inducing GSK3β is a key kinase of Wnt/β-catenin signaling pathway, closely related to tumor cell proliferation and apoptosis, and is a negative regulator of this signaling pathway.^20^ The results of this study showed that the silencing of POLE2 can suppress the Wnt/β-catenin signaling pathway in CRC, participate in the proliferation of CRC cells and promote the occurrence of CRC.

Infinite proliferation and invasion and metastasis are two major characteristics of malignant tumors. Metastasis of tumor cells is the main causes of death in cancer patients.^21^ CRC cells are active, aggressive and rich in blood vessels. It is easy to metastasize and invade, and can invade liver, lymph nodes, lung, brain and other tissues.^22^ The important factor of tumor cell metastasis is the EMT. EMT is critical for tumor cell metastasis.^23^ Epithelial cells with tight adhesion and polarity transform into mobile and nonpolar mesenchymal cells, thereby enhancing the proliferative capacity of tumor cells.^24^ E-cadherin and N-cadherin are closely related to the occurrence of EMT. E-cadherin is an important protein that maintains cell polarity and adhesion connection, and can interact with β-catenin to form complex that play an important role in maintaining the integrity of tissue structure and inhibiting cell migration.^25^ Decreased expression of E-cadherin may be involved in regulating the occurrence and metastasis of CRC.^26^ N-cadherin has the opposite effect to E-cadherin. High expression of N-cadherin can promote tumor cell migration. Wnt/β-catenin signaling pathway is a key pathway to regulate EMT, and the activation pathway can act on the corresponding target genes to induce EMT conversion.^27^

In this study, the deletion of POLE2 gene enhanced the expression of e-cadherin and weakened the expression of N-cadherin, suggesting that POLE2 could promote EMT conversion in CRC cells through the Wnt/β-catenin signaling pathway, thus promoting the metastasis of CRC.

## Conclusion

In conclusion, the expression of POLE2 increased in CRC. Inhibition of POLE2 could increase tumor cells proliferation, migration as well as invasion through Wnt/β-catenin signaling pathway. This may provide potential therapeutic targets for the treatment of CRC.

## Supplementary Material

Supplemental Material

figure S1.jpg

figure S2.jpg

## Data Availability

The datasets used and/or analyzed during the current study are available from the corresponding author on reasonable request.
