# Supplementary material for: POLE2 silencing inhibits the progression of colorectal carcinoma cells via wnt signaling axis
Source: Cancer Biol Ther. 2024 Aug 18;25(1):2392339. doi: 10.1080/15384047.2024.2392339 (PMC11340749; doi:10.1080/15384047.2024.2392339)
Supplement: Supplemental Material [file KCBT_A_2392339_SM8820.docx]

Figure S1. POLE2 is a highly expressed gene in CRC. The mRNA levels of IL12RB1, MRE11 and POT1 in (A) CRC tissues and (B) cell lines were evaluated by RT-qPCR. **p*<0.05, ***p*<0.01, vs NC or FHC group.

Figure S2. Knockdown of POLE2 inhibits cell proliferation, migration and invasion of CRC cells. (A) mRNA and (B) protein expression of POLE2 in HTC116 and SW620 cells after transfection were detected by qPCR and western blot, respectively. (C) CCK-8 and (D-E) colony formation assays were combined to evaluate cell proliferation. (F-H) Transwell assay was performed to determine cell invasion and migration. (I) The protein expression of vimentin, N-cadherin and E-cadherin was evaluated by western blot. ***p*<0.01.
