# Supplementary figures and images for: POLE2 silencing inhibits the progression of colorectal carcinoma cells via wnt signaling axis
Source: Cancer Biol Ther. 2024 Aug 18;25(1):2392339. doi: 10.1080/15384047.2024.2392339 (PMC11340749; doi:10.1080/15384047.2024.2392339)

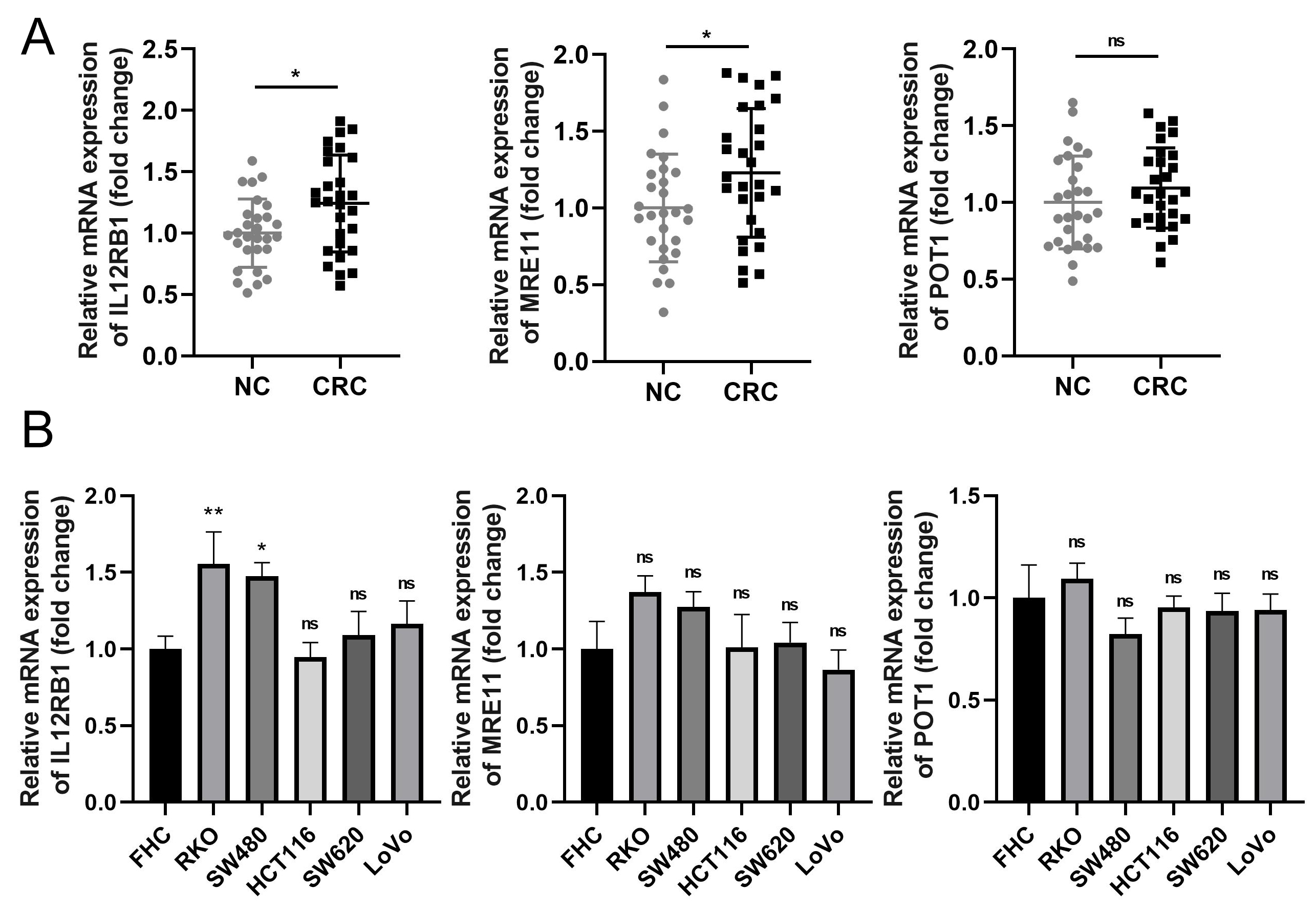

Supplement: figure S1.jpg [file KCBT_A_2392339_SM0111.jpg]

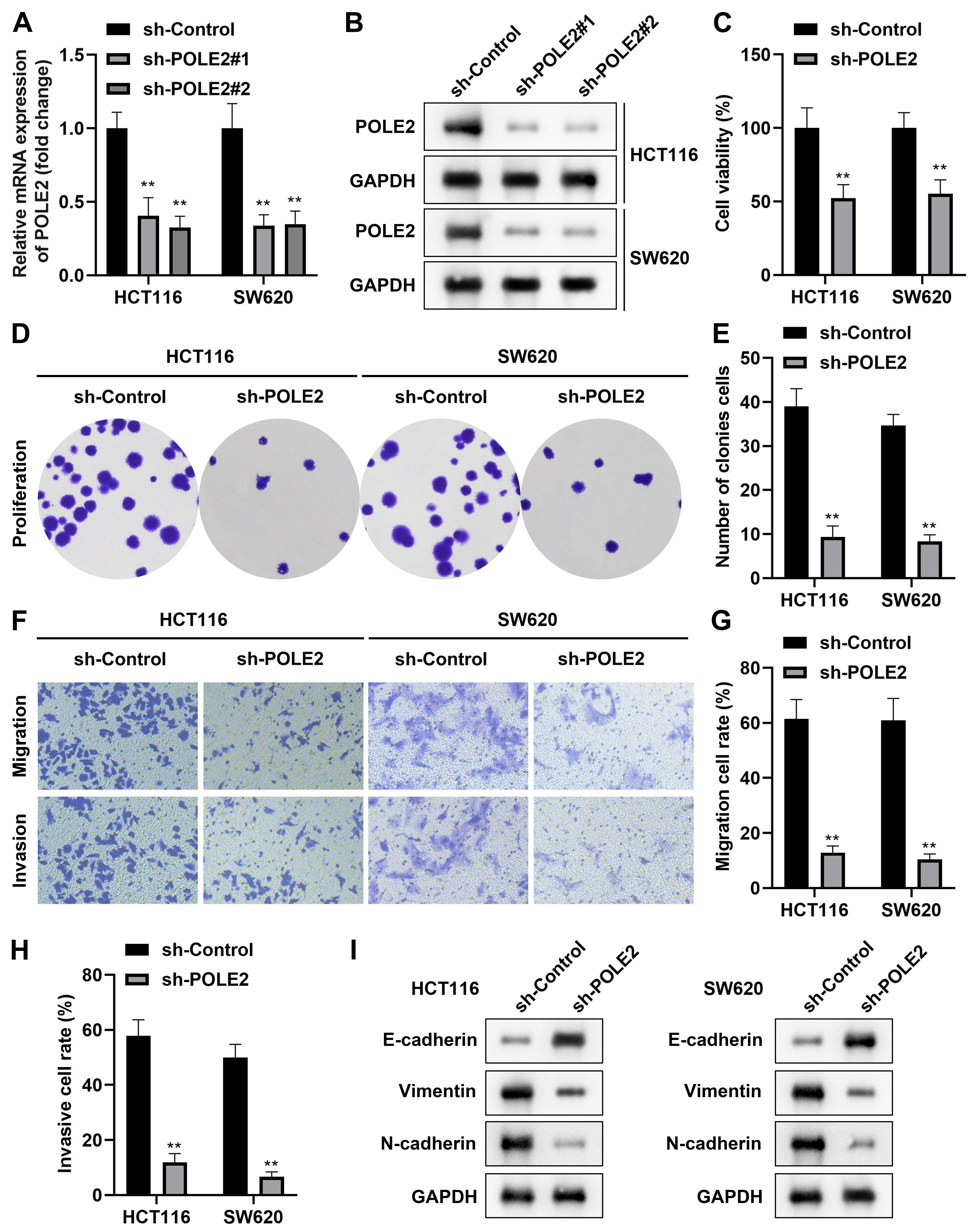

Supplement: figure S2.jpg [file KCBT_A_2392339_SM0110.jpg]
